# Correlation of Coronary Calcium Measured on Conventional Computed Tomography with Coronary Angiography Findings in Lung Transplant Patients

**DOI:** 10.3390/tomography11020011

**Published:** 2025-01-22

**Authors:** Sergio Tapia Concha, Concepción Fariñas-Álvarez, Pedro Muñoz Cacho, José Manuel Cifrian Martínez, Javier Zueco Gil, José Antonio Parra Blanco

**Affiliations:** 1 Department of Radiology, Hospital de Laredo, Av. Derechos Humanos, 40, 39770 Laredo, Spain; sergio.tapia@scsalud.es; 2 Quality Unit, Hospital Universitario Marqués de Valdecilla, Av. de Valdecilla, s/n, 39008 Cantabria, Spain; 3 Instituto de Investigación Valdecilla IDIVAL, 39011 Cantabria, Spain; pedro.munoz@scsalud.es (P.M.C.); josemanuel.cifrian@scsalud.es (J.M.C.M.); 4 Centro de Investigación Biomédica en Red: Enfermedades Infecciosas (CIBERINFEC), 28029 Madrid, Spain; 5 Teaching Department of Primary Care Management, Cantabrian Health Service, IDIVAL, 39011 Cantabria, Spain; 6 Department of Pneumology, Hospital Universitario Marqués de Valdecilla, Av. de Valdecilla, s/n, 39008 Cantabria, Spain; 7 Interventional Cardiology Unit, Department of Cardiology, Hospital Universitario Marqués de Valdecilla, Av. de Valdecilla, s/n, 39008 Cantabria, Spain; 8 Department of Radiology, Hospital Universitario Marqués de Valdecilla, Av. de Valdecilla, s/n, 39008 Cantabria, Spain; 9 School of Medicine, Universidad de Cantabria (UNICAN), 39011 Cantabria, Spain

**Keywords:** lung transplantation, coronary artery disease, coronary calcium, coronary calcium on computed tomography, coronary angiography

## Abstract

**Introduction and objective:** The pre-transplant protocol for lung transplant candidates includes a chest CT scan to assess disease progression and often coronary angiography (CA) to rule out coronary artery disease (CAD). Coronary artery calcium is commonly observed in these pre-transplant CT scans. This study aims to evaluate the relationship between coronary calcium detected on CT and findings from CA to determine whether calcium presence could serve as an additional criterion for selecting patients for CA. **Material and Methods**: We included 252 consecutive lung transplant patients who had both a CT scan and CA within 365 days of each other. Coronary calcium quantification was performed using artery-based, segment artery-based, and visual assessment methods. CA findings were classified by stenosis severity: ≤20%, 21–70%, and >70%. **Results:** This study showed very high concordance (kappa = 0.896; 95% CI: 0.843–0.948) between the three methods, especially in distinguishing patients without and with coronary calcium (kappa = 1.000; 95% CI: 0.929–1.071). ROC analysis identified the absence of coronary calcium as the best cutoff to differentiate patients with ≤20% stenosis from those with >21%, with a sensitivity of 73.5%, specificity of 55.7%, PPV of 28.5%, and NPV of 90%. Only 11 patients (8.7%) without coronary calcium had stenosis of 21–70%, and only 2 (1.6%) had stenosis > 70%. **Conclusions:** The visual assessment method yielded results similar to the other two quantification methods. The absence of coronary calcium in pre-transplant CT may be a useful criterion for selecting patients for CA.

## 1. Introduction

Lung transplant candidates, due to age (lung transplants are increasingly performed in older patients), the presence of one or more cardiovascular risk factors, and underlying lung disease, have a high risk of developing coronary artery disease (CAD) [1]. This risk is particularly significant in patients with advanced-stage pulmonary fibrosis [2] and emphysema/COPD (3). For reasons not yet fully understood, patients with pulmonary fibrosis and emphysema/COPD have a higher risk of CAD compared to those with other pulmonary conditions [2,3,4].

Estimating the prevalence of CAD in this population is challenging due to the lack of a consistent coronary angiography (CA) threshold to define the disease (e.g., stenosis greater than 50% or greater than 70%). However, it is estimated that the prevalence of CAD in these patients ranges between 5% and 24% and around 11% when the threshold is set at >70% in a major coronary artery or >50% in the left main coronary artery [5,6]. Advances in percutaneous and vascular revascularization techniques have significantly improved the prognosis of these patients to the point that recent studies suggest that those patients with mild to moderate CAD or a history of previous revascularization do not experience an increase in perioperative complications or a reduction in survival compared to patients without CAD [6,7,8]. Despite these advances, CAD remains a relative contraindication for lung transplantation [9], and for this reason, many centers include CA in the pre-transplant protocol for patients over 40 to 45 years of age [10,11]. However, CA is an invasive procedure that is not without risks. In the study by West et al. [12], 3.3% of patients experienced complications attributable to this procedure. As a consequence of this situation and given the lack of an established protocol, several authors propose performing non-invasive tests prior to CA, such as coronary CT angiography, Dobutamine stress echocardiography, Stress cardiac MRI, or Myocardial perfusion imaging with single-photon emission computed tomography (SPECT). According to these protocols, coronary angiography should only be indicated in cases where there is evidence of obstruction [1,4].

Coronary calcium is commonly detected on chest CT scans during pre-transplant evaluations. Numerous cohort studies have established an association between coronary calcium and an increased risk of CAD, cardiovascular events, and mortality [13]. This association has led the American College of Cardiology and the American Heart Association to include coronary calcium quantification in their guidelines for assessing cardiovascular risk in low- to intermediate-risk individuals [14]. Most studies use the Agatston method to quantify coronary calcium [15]; while widely used, this method requires specialized software [16]. Recently, alternative methods based on ordinal [17,18,19] or qualitative scales [18], applicable to both low-dose and conventional CT scans [16,17,18,19,20], have shown a strong correlation with the Agatston score [12,21,22].

In liver transplant candidates, several studies [12,23,24] have evaluated the correlation between coronary calcium (quantified using the Agatston method, either with gated or non-gated techniques) and angiographic findings. These studies concluded that coronary calcium levels correlate with the degree of stenosis observed in CA and that the absence of calcium effectively rules out stenosis greater than 50%. According to these authors, using these parameters could have avoided up to 28% of the number of CAs performed without missing significant CAD [12]. In the field of lung transplantation, to our knowledge, this is the first study to analyze the relationship between the amount of coronary calcium detected on a chest CT scan conducted as part of the pre-transplant protocol and CA findings. Our objective, like that of the authors previously mentioned [12,23,24], is to clarify the relationship between the amount of calcium detected on CT during the pre-transplant protocol and the findings on CA and to assess whether these results could be considered to select patients in whom CA could potentially be avoided without compromising the detection of significant CAD.

## 2. Materials and Methods

### 2.1. Study Design

This retrospective cohort study was conducted at a tertiary referral teaching hospital and included all lung transplant patients from January 2008 to October 2018, totaling 416 patients. All patients had a minimum follow-up period of three years. Information on transplant indications, as well as the dates of CT scans, coronary angiographies (CA), and lung transplants, was extracted from the patients’ medical records. Only recipients with both CT and CA data obtained within a 365-day interval were included in this study; patients with prior coronary stents were also excluded due to potential interference with coronary calcium quantification [11].

The pre-lung transplant protocol at our hospital involved a medical history, physical examination, laboratory tests, chest CT, right heart catheterization, and CA for patients over 50 years as well as those with a history of smoking (>10 pack-years), cardiovascular risk factors, a history of ischemic heart disease, or left ventricular dysfunction identified by echocardiography.

Coronary calcium quantification on CT scans was conducted by two radiologists with expertise in thoracic radiology and cardiac CT, and, in cases of uncertainty, the final decision was reached by consensus. The reviewers were blinded to clinical data, risk factors, and CA findings during the assessment of coronary calcium. All assessments were conducted using multidetector CT, with images evaluated at a window width of 1000 and a level of approximately −100.

We used three quantification methods: the ordinal artery-based scoring proposed by Shemesh et al. [17], the ordinal segmented artery-based method, and the visual ºassessment method described by Chiles J et al. [18].

According to the artery-based scoring method [17], coronary calcium in the four main arteries (left coronary trunk, anterior descending, circumflex, and right coronary arteries) was quantified on a scale of 0 to 3, in which 0 is no calcification, one is mild calcification, two is moderate calcification, and three severe calcifications. Calcification was considered mild when less than one-third of the length of the vessel was calcified; one to two-thirds were moderate, and more than two-thirds were severe. By this method, the total amount of coronary calcium can range from 0 to 12. For the purposes of the analysis, 0 was classified as no calcification, 1–3 mild calcification, 4–6 moderate calcification, and 7–12 severe calcification.

In the segmented artery-based method [18], the left main coronary, left anterior descending, left circumflex, and right coronary were evaluated. The left anterior descending, left circumflex, and right coronary were divided into three segments: proximal, middle, and distal. Coronary calcium was classified in each as absent, mild, moderate, or severe on a scale of 0–3, in which 0 was the absence of calcium, 1 was small flecks of calcium within a segment, 2 was calcification greater than mild and less than severe, and 3 was the observation of continuous calcification within the segment. Using this method, the total sum of the coronary calcium scores in the four vessels can range between 0 and 30. For study purposes, 0 was classified as no calcification, 1–5 mild, 6–11 moderate, and 12–30 severe.

According to the visual assessment method [18], coronary calcium was classified as absent when none was visible to the naked eye and mild, moderate, or severe, depending on the amount of calcium observed in the entire coronary tree.

CT scan quality was evaluated as adequate, suboptimal (patient of technical factors compromised image quality), or inadequate (patient or technical factors precluded assessment of CAC) [18].

CA findings were obtained from hemodynamic reports and were divided according to the system of Kandahar et al. [11] into the following categories: stenoses ≤20%, 21–70%, and >70%. CA findings were placed into one of these categories when at least one main artery had this degree of stenosis.

This study adhered to the guidelines of the Declaration of Helsinki and received approval from the institutional Ethics Committee. Due to the nature of this study, patient consent was waived, as there was no alteration to clinical or therapeutic approaches.

### 2.2. Statistical Analysis

Statistical analysis was performed using a two-tailed χ2 test, Fisher’s exact test, Student’s *t*-test, ANOVA test, or the Mann–Whitney test, as appropriate in each case. For m × n tables, Fisher’s exact test was estimated using the Monte Carlo method.

Kappa statistics with 95% confidence intervals (95% CIs) were used to assess the concordance between the three methods of quantifying the calcium score obtained from CT scans. Sensitivity, specificity, positive predictive value (PPV), and negative predictive value (NPV) were calculated using coronary angiography results as the reference test; the 95% CIs were calculated as exact binomial confidence intervals. The diagnostic accuracy of the different methods of quantifying coronary calcium was evaluated by constructing ROC (receiver operating characteristic) curve areas computed using the trapezoidal rule.

A *p*-value of less than 0.05 (two-sided) was considered statistically significant. Calculations were performed with IBM SPSS Statistics (v. 28.0, IBM Corp, Armonk, NY, USA) and Stata statistical software (Release 11.0, Stata Corporation, College Station, TX, USA).

## 3. Results

**Figure 1:**
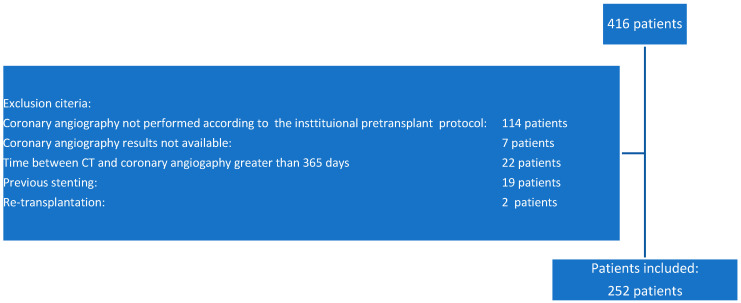
Flow diagram depicting patient selection.

Of the 416 lung transplant patients, 252 had both a CT scan and coronary angiography performed within an interval of less than 365 days and constituted the study base. Of the 164 excluded patients, 114 did not have a coronary angiography performed according to our own institution protocol; in 7 cases, the coronary angiography results were unavailable; in 22 cases, the time between the CT scan and coronary angiography exceeded 365 days, and 2 patients were excluded due to undergoing a lung retransplant (Figure 1).

All CT and CA studies were performed before transplantation. The mean interval between the CT scan and coronary angiography in the 225 patients was 109.7 days, with an SD of 106.2 and a range of 4–356.

All the studies were conducted using multidetector CT scanners (2–128 detectors), with 98.8% of scans having a slice thickness of ≤5 mm (see Appendix A). In total, 147 studies (58.7%) were performed without intravenous contrast agents. The quality was deemed adequate in 88.5% of the studies and suboptimal in 11.5%; no studies were classified as inadequate. We found a statistically significant association between study quality and the non-administration of intravenous contrast agents (*p* = 0.008), as well as a trend toward higher quality studies with CT scans performed using a higher number of detectors and a lower slice thickness (*p* = 0.007 and *p* = 0.012, respectively).

The degree of concordance between the three coronary calcium quantification methods was very good, with a kappa index of 0.896 (95% CI 0.843–0.948), which was statistically significant (*p* < 0.001). The best concordance was found between the ordinal segmented artery-based method and the visual assessment method (kappa 0.954 [95% CI 0.862–1.046]). An analysis of the level of concordance in each category between the three methods showed that the concordance was at its maximum for diagnoses without coronary calcium (kappa 1.000; 95% CI 0.929–1.071); concordance was very good for mild calcification (kappa 0.952; 95% CI 0.854–0.997), good for moderate calcification (kappa 0.711; 95% CI 0.604–0.746), and moderate for severe calcification (kappa 0.542; 95% CI 0.455–0.598).

Furthermore, 126 of the 252 patients were observed to have coronary calcium according to their CT scan. Among them, the majority presented calcification that was considered mild (between 68.2% and 73.8%, depending on the quantification method used), moderate (between 18.3% and 27.0%), and severe (between 4.8% and 7.9%). See Table 1.

Compared with patients without coronary calcium (see Table 2), those with coronary calcium were older (*p* = 0.005), more frequently male (*p* = 0.001), and had a higher number of coronary risk factors (*p* = 0.001), including smoking habits, hypercholesterolemia, and a BMI > 30 (*p* < 0.05). Among both groups, both those with and without coronary calcification, the most common indications for lung transplantation were diffuse Interstitial Lung Disease (ILD) and emphysema/Chronic Obstructive Pulmonary Disease (COPD), with no statistically significant differences observed (Table 3).

Using the visual assessment method as a reference (Table 4), we found no statistically significant differences in age, sex, or cardiovascular risk factors between patients with mild, moderate, or severe calcification. However, a higher degree of calcification was associated with an older median age, a greater proportion of male patients, and a higher number of cardiovascular risk factors. In total, 95.7% of patients with moderate calcification and 90% of patients with severe calcification underwent transplantation due to diffuse ILD or emphysema/COPD (see Table 5).

In 203 patients (80.6%), their CA revealed no significant changes (stenosis ≤ 20%). In 41 patients (16.3%), stenosis ranged from 21% to 70%, and in 8 patients (3.2%), it was greater than 70%. Moderate and severe stenoses were more common among older patients, men, and those with a higher number of cardiovascular risk factors (see Table 6). A total of 88% of patients with moderate stenosis, according to their CA, and 87.5% with severe stenosis underwent transplantation because of diffuse ILD (UIP) or emphysema/COPD (Table 7).

As shown in Table 8, coronary calcium was observed most frequently in patients with stenosis > 20% using all three methods. Among the eight patients with stenosis > 70%, two did not have calcifications according to their CT, and one showed mild calcification.

**Figure 2:**
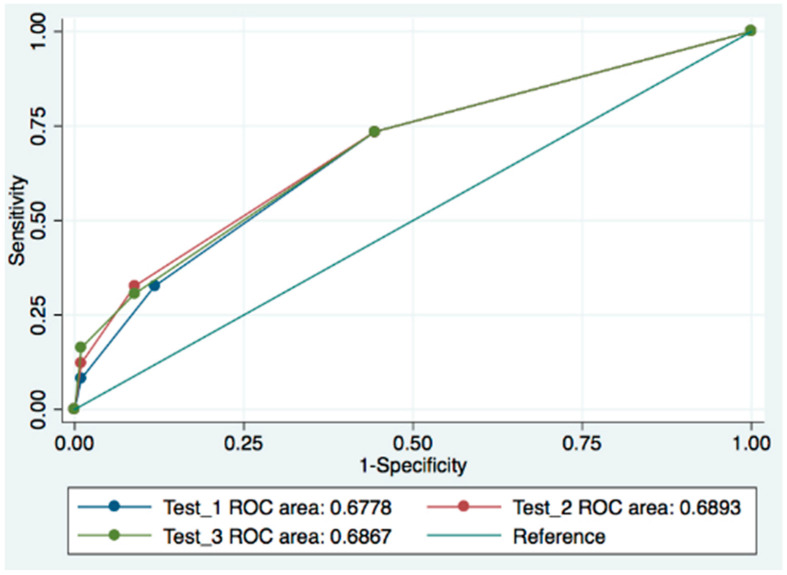
Comparison of the ROC curves of the three methods of calcium quantification according to CT findings with coronary angiography. Test 1. Artery-based method; Test 2: segment artery-based method; Test 3: visual assessment method.

To compare the coronary calcium and CA findings, given the low number of patients with stenosis > 70%, we grouped the results of the CA findings into the following two categories: stenosis ≤ 20% and >21%. ROC analysis showed similar areas under the curve for all three methods (Figure 2), with no statistically significant differences between them. The results were as follows: 0.678 (95% CI: 0.597–0.758) for the artery-based method, 0.689 (95% CI: 0.608–0.771) for the segment artery-based method, and 0.687 (95% CI: 0.606–0.768) for the visual assessment method (see Figure 2).

ROC analysis demonstrated that the cutoff point distinguishing “no calcification” from “presence of calcification” (mild, moderate, or severe) optimized sensitivity and specificity for detecting stenosis ≤ 20% and >21%. Using this threshold, the sensitivity was 73.5%, and the specificity was 55.7%. PPV and NPV were 28.6% and 89.7%, respectively (Table 9). Among the 13 patients (10.3%) with no calcification and stenosis > 20%, 2 had stenosis greater than 70%, and the remaining patients had stenosis between 21 and 70%, according to their CA.

## 4. Discussion

Although the Agatston score is widely regarded as the gold standard for coronary calcium quantification, several studies have shown that coronary calcium assessed through conventional or low-dose CT scans correlates closely with data obtained using the Agatston method [12,17,18,19,20,21,22,23,24,25,26,27,28,29].

Lung transplant patients often undergo multiple CT scans throughout the course of their disease. Combined with the fact that many of these patients also undergo CA as part of the transplant protocol, this population is ideal for examining the relationship between coronary calcium detected on CT scans and CA results.

This study spans a 10-year period and includes CT scans performed with various detector configurations, slice thicknesses, and both with and without intravenous contrast agents. Despite these variations, 88.5% of the CT scans were deemed adequate for assessment. Our study confirms that equipment with a higher number of detectors, lower slice thickness [22,25], and non-use of contrast agents enhances the accuracy of coronary calcium quantification and improves the quality of findings.

In our study, all three methods of calcium quantification—artery-based, segment artery-based, and visual assessment—demonstrated similar diagnostic validity, with only minor differences in the ROC area. Consequently, these methods can be used interchangeably. We selected the visual assessment method for its simplicity and speed.

As stated [1,4], CAD is a frequent finding in candidates for lung transplantation, and as a consequence, coronary calcium as a marker of CAD is commonly observed in the pre-transplantation CT scans conducted as part of the evaluation protocol. In our study, up to 50% of patients showed coronary calcification, classified as mild in 73.8% of cases. Compared to patients with mild calcification, those with moderate or severe calcification tended to be older, predominantly male, had more risk factors, and over 90% had an underlying interstitial lung disease (ILD), followed by emphysema/COPD.

Regarding coronary angiography findings, most patients (80.6%) showed no significant abnormalities (stenosis less than 20%), while 3.2% exhibited stenosis greater than 70%. This proportion is lower than reported by authors like Jones RM et al. [5] and Lusenbrink E et al. [6], whose studies indicate about 11%. This difference may be attributed to our exclusion of patients with prior stents. Consistent with previous studies [5,10,26,27,28,29,30], patients with more severe stenosis were generally older, predominantly male, had multiple cardiovascular risk factors, and had ILD as their underlying end-stage pulmonary disease. In six of the eight patients with stenosis > 70%, pulmonary fibrosis was the underlying cause, with emphysema/COPD in one case.

In the context of liver transplantation, two studies [23,24] using the Agatston score method identified associations between scores above 400 and 250, respectively, and the presence of coronary stenosis. West et al. [12] found a relationship between an Agatston score above 251, a coronary calcium Weston score above 6, and >50% stenosis, according to CA. These authors also reported that a Weston score < 2 excludes the presence of obstructive coronary disease with 100% sensitivity and a 100% NPV [12]. In contrast, our study did not identify a specific coronary calcium threshold that could reliably predict moderate or significant stenosis on coronary angiography, suggesting that CA should be mandatory. However, we observed that the absence of coronary calcium on CT scans, as reported by this and other authors [31,32], allows for the identification of a significant subgroup of patients with a very low likelihood of significant stenosis, for whom coronary angiography may not be necessary (NPV 90%). Among the 126 patients without coronary calcium, only 13 showed stenosis > 21%, and just 2 (1.6%) had stenosis > 70%. These results align with those of Schuhbaeck et al. [31], who found that among 1032 patients with an Agatston calcium score of 0, only 2.6% had at least one stenosis ≥ 75%. They are also close to Haberl R et al. [32] findings, with an incidence of significant stenosis in patients with a calcium score of 0 of less than 1%. The amount of coronary calcium, as observed by O’Rourke RA et al. [33], is highly sensitive (91%) for detecting ≥ 50% stenosis but has limited specificity (49%). Our results, with a sensitivity of 73.5% and a specificity of 55.7%, follow this trend, although with slightly lower percentages. This could be explained by the difference in the cutoff point used: ≥21% stenosis compared to the ≥50% threshold considered by O’Rourke and colleagues.

### Limitations

Our study has several limitations. First, a substantial number of studies were conducted with CT scans using detectors with fewer than 64 channels, variable slice thicknesses, and sometimes with contrast agents, complicating the assessment of coronary calcium, especially for mild calcifications and plaques that may be missed due to partial volume effects or motion artifacts. The lack of a reference standard, such as the Agatston score, is also a limitation, as this score is not routinely included in the pre-lung transplant protocol at many centers, including ours. The exclusion of prior stenting patients may have introduced a potential bias in the results of patients with severe coronary disease, and, lastly, the small number of patients with moderate-to-severe stenosis limited our ability to thoroughly analyze the significance of these findings in conventional CT scans performed during the pre-transplant period. A larger patient population might yield more definitive results.

## 5. Conclusions

The strong correlation among the three coronary calcium quantification methods suggests that any of them can effectively be used to evaluate coronary calcium on CT scans performed in lung transplantation candidates during the preoperative period. In this regard, the visual assessment method is particularly advantageous due to its speed and ease of use. Only 1.6% of patients without coronary calcium present stenosis greater than 70%. This information, combined with factors such as patient age, sex, risk factors, and the indication for transplantation, could be taken into account as a parameter to select patients for other noninvasive methods or CA. Based on our findings, we recommend including the degree of calcification, at least as assessed by the visual method, in the CT report as part of the pre-lung transplant evaluation protocol.

## Figures and Tables

**Table 1:** Quantification of coronary calcium in the three methods.

	Coronary Calcium Quantification Method		
Coronary Calcium Quantification	Artery-Based *n* = 126 (%)	Segment-Based *n* = 126 (%)	Visual Assessment *n* = 126 (%)
Mild calcification	86 (68.2)	92 (73.0)	93 (73.8)
Moderate calcification	34 (27.0)	26 (20.7)	23 (18.3)
Severe calcification	6 (4.8)	8 (6.3)	10 (7.9)

**Table 2:** Characteristics of this study population according to the presence or absence of coronary calcium on CT scans.

	Patients Without Coronary Calcium	Patients with Coronary Calcium	*p*-Value
	*n* = 126 (%)	*n* = 126 (%)	
Median age (SD) years	56 (7.5)	59 (4.8)	<0.005
Men	57 (7.1)	60 (4.6)	0.014
Women	56 (8.0)	59 (5.5)	0.092
Gender			
Male	66 (53.4)	102 (81.0)	<0.001
Female	60 (47.6)	24 (19.0)	
Cardiovascular risk factors	105 (83.3)	122 (96.8)	<0.001
Tobacco consumption	87 (69.0)	108 (85.7)	0.002
Arterial hypertension	21 (16.7)	36 (28.6)	0.024
Hypercholesterolemia	29 (23.0)	48 (38.1)	0.009
Diabetes mellitus	13 (10.6)	21(16.7)	0.14
BMI > 30	36 (28.6)	53 (42.1)	0.025

BMI > 30: body mass index greater than 30.

**Table 3:** Reasons for lung transplantation, according to the presence or absence of coronary calcification obtained from a CT scan, are estimated using the visual assessment method.

Causes of Transplantation	Patients Without Coronary Calcium	Patients with Coronary Calcium	*p*-Value
	*n* = 126 (%)	*n* = 126 (%)	
ILD	53 (42.1)	58 (46.0)	0.534
UIP	38 (30.2)	45 (35.7)	0.354
NSIP	4 (3.2)	6 (4.8)	0.518
Lymphangioleiomyomatosis	3 (2.4)	0 (0.0)	0.081
Histiocytosis	3 (2.4)	2 (1.6)	0.651
Sarcoidosis	3 (2.4)	2 (1.6)	0.651
Other	2 (1.6)	3 (2.4)	0.651
Emphysema/COPD	45 (35.7)	50 (39.7)	0.513
Emphysema/COPD	34 (27.0)	44 (34.9)	0.176
Alpha 1 antitrypsin deficiency	11 (8.7)	6 (4.8))	0.166
Bronchiectasis/Cystic fibrosis	9 (7.1)	3 (2.4)	0.08
Bronchiectasis	6 (4.8)	3 (2.4)	0.308
Cystic fibrosis	3 (2.4)	0 (0.0)	0.081
Pulmonary hypertension	1 (0.8)	0 (0.0)	0.315
Secondary	1 (0.8)	0 (0.0)	0.315
Bronchiolitis obliterans	2 (1.6)	1 (0.8)	0.561
Occupational lung disease	12 (9.5)	10 (7.9)	0.653
Pneumoconiosis	2 (1.6)	6 (4.8)	0.15
HN/EAA	10 (7.9)	4 (3.2)	0.104
Connective tissue disease	4 (3.2)	3 (2.4)	0.701
Rheumatoid arthritis	1 (0.8)	2 (1.6)	0.561
Scleroderma	2 (1.6)	1 (0.8)	0.561
Churg Strauss	1 (0.8)	0 (0.0)	0.315
Other	0 (0.0)	1 (0.8)	0.315

ILD: interstitial lung disease. UIP: usual interstitial pneumonia. NSIP: non-specific interstitial pneumonia. COPD: chronic obstructive pulmonary disease. HN/EAA: hypersensitivity pneumonitis/extrinsic allergic alveolitis.

**Table 4:** Characteristics of the study population according to the degree of coronary calcification estimated using the visual assessment method.

	Mild Calcification	Moderate Calcification	Severe Calcification	*p*-Value
	*n* = 93 (73.8)	*n* = 23 (18.3)	*n* = 10 (7.9)	
Median age (SD) years	58 (6.4)	59 (5.7)	60 (4.2)	0.299
Men	59 (6.3)	59 (5.7)	60 (3.9)	0.611
Women	56 (6.4)	63 (4.8)	-	0.032
Gender				
Male	72 (77.4)	21 (91.3)	9 (90.0)	0.098
Female	21 (22.6)	2 (8.7)	1 (10.0)	
Cardiovascular risk factors	89 (95.7)	23 (100)	10 (100)	0.107
Tobacco consumption	79 (84.9)	20 (87.0)	9 (90.0)	0.735
Arterial hypertension	32 (34.4)	7 (30.4)	4 (40.0)	0.311
Hypercholesterolemia	44 (47.3)	11 (47.8)	4 (40.0)	0.322
Diabetes mellitus	21 (22.6)	7 (30.4)	1 (10.0)	0.162
BMI > 30	49 (52.7)	12 (52.2)	1(10.0)	0.069

BMI > 30: body mass index greater than 30.

**Table 5:** Reasons for lung transplantation, according to the degree of coronary calcification obtained from a CT scan, estimated using the visual assessment method.

	Mild Calcification	Moderate Calcification	Severe Calcification
	*n* = 93 (73.8)	*n* = 23 (18.3)	*n* = 10 (7.9)
Causes of transplantation			
ILD	37 (39.8)	15 (65.2)	6 (60.0)
UIP	28 (30.1)	12 (52.2)	5 (50.0)
NSIP	4 (4.3)	2 (8.7)	0 (0.0)
Histiocytosis	1 (1.1)	1 (4.3)	0 (0.0)
Sarcoidosis	2 (2.1)	0 (0.0)	0 (0.0)
Other	2 (2.1)	0 (0.0)	1 (10.0)
Emphysema/COPD	40 (43.0)	7 (30.4)	3 (30.0)
Emphysema/COPD	35 (37.6)	7 (30.4)	2 (20.0)
Alpha 1 antitrypsin deficiency	5 (5.4)	0 (0.0)	1 (10.0)
Bronchiectasis/Cystic fibrosis	2 (2.1)	1 (4.3)	0 (0.0)
Bronchiectasis	2 (2.1)	1 (4.3)	0 (0.0)
Bronchiolitis obliterans	1 (1.1)	0 (0.0)	0 (0’0)
Occupational lung disease	9 (9.7)	0 (0.0)	1 (10.0)
Pneumoconiosis	6 (6.5)	0 (0.0)	0 (0.0)
HN/EAA	3 (3.2)	0 (0.0)	1 (10.0)
Connective tissue disease	3 (3.2)	0 (0.0)	0 (0.0)
Rheumatoid arthritis	2 (2.1)	0 (0.0)	0 (0.0)
Scleroderma	1 (1.1)	0 (0.0)	0 (0.0)
Other	1 (1.1)	0 (0.0)	0 (0.0)

ILD: interstitial lung disease. UIP: usual interstitial pneumonia. NSIP: non-specific interstitial pneumonia. COPD: chronic obstructive pulmonary disease. HN/EAA: hypersensitivity pneumonitis/extrinsic allergic alveolitis.

**Table 6:** Characteristics of the study population according to coronary angiography findings.

	Coronary Angiography Findings. *n* = 252 Patients (%)			
	Stenosis ≤ 20%	Stenosis 21–70%	Stenosis ≥ 70%	*p*-Value
	*n* = 203 (80.6)	*n* = 41 (16.3)	*n* = 8 (3.2)	
Median age (SD) years	57.1 (6.7; 25–70)	60.5(5.1;46–68)	61 (2.8;56–66)	0.003
Men	57.6 (6.0; 31–70)	61.4 (4.4; 51–68)	60.3 (2.1;35–62)	0.002
Women	56.4 (7.6; 25–66)	57.2 (6.5; 46–68)	66	0.43
Gender				
Male	129 (63.5)	32 (78.0)	7 (87.5)	0.103
Female	74 (36.5)	9 (22.0)	1 (12.5)	
Cardiovascular risk factors	179 (88.2)	40 (97.6)	8 (100)	0.118
Tobacco consumption	152(74.9)	36 (87.8)	7 (87.5)	0.163
Arterial hypertension	44 (21.7)	9 (22.0)	4 (50.0)	0.187
Hypercholesterolemia	55 (27.1)	18 (43.9)	4 (50.0)	0.044
Diabetes mellitus	24 (11.8)	9 (22.0)	1 (12.5)	0.212
BMI > 30	71 (35.0)	15 (36.6)	3 (37.5)	0.8

BMI > 30: body mass index greater than 30.

**Table 7:** Reasons for lung transplantation according to the degree of coronary stenosis reported from coronary angiography.

	Coronary Angiography Findings. *n* = 252 Patients (%)		
	Stenosis ≤ 20%	Stenosis 21–70%	Stenosis ≥ 70%
	*n* = 203 (80.6)	*n* = 41 (16.3)	*n* = 8 (3.2)
Causes of transplantation			
ILD	85 (41.9)	20 (48.8)	6 (75.0)
UIP	62 (30.5)	16 (39.0)	5 (62.5)
NSIP	10 (4.9)	0 (0.0)	0 (0.0)
Lymphangioleiomyomatosis	3 (1.5)	0 (0.0)	0 (0.0)
Histiocytosis	4 (2.0)	0 (0.0)	1 (12.5)
Sarcoidosis	5 (2.5)	0 (0.0)	0 (0.0)
Other	1 (0.5)	4 (9.8)	0 (0.0)
Emphysema/COPD	78 (38.4)	16 (39.0)	1 (12.5)
Emphysema/COPD	62 (30.5)	16 (39.0)	0 (0.0)
Alpha 1 antitrypsin deficiency	16 (7.9)	0 (0.0)	1 (12.5)
Bronchiectasis/Cystic fibrosis	10 (4.9)	2 (4.9)	0 (0.0)
Bronchiectasis	7 (3.4)	2 (4.9)	0 (0.0)
Cystic fibrosis	3 (1.5)	0 (0.0)	0 (0.0)
Pulmonary hypertension	1 (0.5)	0 (0.0)	0 (0.0)
Secondary	1 (0.5)	0 (0.0)	0 (0.0)
Bronchiolitis obliterans	3 (1.5)	0 (0.0)	0 (0’0)
Occupational lung disease	19 (9.3)	3 (7.3)	1 (12.5)
Pneumoconiosis	5 (2.5)	2 (4.9)	1 (12.5)
HN/EAA	14 (6.9)	1 (2.4)	0 (0.0)
Connective tissue disease	7 (3.4)	0 (0.0)	0 (0.0)
Rheumatoid arthritis	3 (1.5)	0 (0.0)	0 (0.0)
Scleroderma	2 (1.0)	0 (0.0)	0 (0.0)
Churg Strauss	1 (0.5)	0 (0.0)	0 (0.0)

ILD: interstitial lung disease. UIP: usual interstitial pneumonia. NSIP: non-specific interstitial pneumonia. COPD: chronic obstructive pulmonary disease. HN/EAA: hypersensitivity pneumonitis/extrinsic allergic alveolitis.

**Table 8:** Comparison of coronary calcium using the three CT quantification methods with coronary angiography results.

	Coronary Arteriography. *n* = 252 Patients (%)		
Artery-based *n* = 252 patients (%)	Stenosis ≤ 20% *n* = 203 (80.6)	Stenosis 21–70% *n* = 41 (16.3)	Stenosis > 70% *n* = 8 (3.2)
No coronary calcium *n* = 126 (50.0)	113 (55.7)	11 (26.8)	2 (25.0)
Mild calcification *n* = 86 (34.1)	66 (32.5)	19 (46.3)	1 (12.5)
Moderate calcification *n* = 34 (13.5)	22 (10.8)	8 (19.5)	4 (50.0)
Severe calcification *n* = 6 (2.4)	2 (1.0)	3 (7.3)	1 (12.5)
Segment artery-based *n* = 252 patients (%)			
No coronary calcium *n* = 126 (50.0)	113 (55.7)	11 (26.8)	2 (25)
Mild calcification *n* = 92 (36.5)	72 (35.5)	19 (46.3)	1 (12.5)
Moderate calcification *n* = 26 (10.3)	16 (7.9)	7 (17.1)	3 (37.5)
Severe calcification *n* = 8 (3.2)	2 (1.0)	4 (9.8)	2 (25)
Visual assessment *n* = 252 patients (%)			
No coronary calcium *n* = 126 (50.0)	113 (55.7)	11 (26.8)	2 (25.0)
Mild calcification *n* = 93 (36.9)	72 (35.5)	20 (48.8)	1 (12.5)
Moderate calcification *n* = 23 (9.1)	16 (7.9)	4 (9.8)	3 (37.5)
Severe calcification *n* = 10 (4.0)	2 (1.0)	6 (14.6)	2 (25.0)

**Table 9:** Comparison of sensitivity, specificity, PPV, and NPV for the different cutoff points for the three methods of coronary calcium quantification obtained from CT scans with the results of coronary angiography in 252 lung transplant patients.

Methods of Coronary Calcium Quantification in CT (Cut-Off Points According to the Presence of Calcification).	CG ≤ 20%	CG ≥ 21%	Sensibility (%) (95% IC)	Specificity (%)	PPV (%)	NPV (%)	ROC Area (95% IC)
	TN/Total	TP/Total		(95% CI)	(95% IC)	(95% IC)	
Artery based (cutting points)							
No/mild-moderate-severe	113/203	36/49 16/49	73.5 (61.1–85.8)	55.7 (48.8–62.5)	28.6 (20.7–36.5)	89.7 (84.4–95.0)	0.678 (0.597–0.758)
No-mild/moderate-severe	179/203	4/49	32.7 (19.5–45.8)	88.2 (83.7–92.6)	40.0 (24.8–55.2)	84.4 (79.5–89.3)	
No-mild-moderate/severe	201/203		8.2 (5.0–15.8)	99.1 (97.6–100)	66.7 (29.0–100)	81.7 (76.9–86.5)	
Segment artery based (cutting points)							
No/mild-moderate-severe	113/203	36/49	73.5 (61.1–85.8)	55.7 (48.8–62.5)	28.6 (20.7–36.5)	89.7 (84.4–95.0)	0.689 (0.608–0.771)
No-mild/moderate-severe	185/203	16/49	32.7 (19.5–45.8)	91.1 (87.2–95.0)	47.1 (30.3–63.8)	84.9 (80.1–89.6)	
No-mild-moderate/severe	201/203	6/49	12.2 (3.1–21.4)	99.0 (97.6–100)	75.0 (45.0–100)	82.4 (77.6–87.2)	
Visual assessment (cutting points)							
No/mild-moderate-severe	113/203	36/49	73.5 (61.1–85.8)	55.7 (48.8–62.5)	28.6 (20.7–36.5)	89.7 (84.4–95.0)	0.687 (0.606–0.768)
No-mild/moderate-severe	185/203	15/49	30.7 (17.7–43.5)	91.1 (87.2–95.0)	45.5 (28.5–62.4)	84.5 (79.7–89.3)	
No-mild-moderate/severe	201/203	8/49	16.3 (6.0–26.7)	99.0 (97.6–100)	80.0 (55.2–100)	83.1 (78.3–87.8)	

CG ≤ 20%: coronary angiography with stenosis ≤ 20%; CG ≥ 21%: coronary angiography with stenosis ≥ 21%; TP: true positive; TN: true negative; CI: confidence interval; PPV: positive predictive value; NPV: negative predictive value.

## Data Availability

Dataset available on request from the corresponding authors.

## Appendix A

**Table A1:** CT study characteristics.

	Total Sample	Suboptimal Quality	Adequate Quality	*p*-Value
	*n* = 252	*n* = 29 (%)	*n* = 223 (%)	
N.° of detectors	*n* = 215 (%)			
2	100 (46.5)	19 (73.1)	81 (42.9)	0.067
10	1 (0.5)	0 (0.0)	1 (0.5)	
16	24 (11.2)	2 (7.7)	22 (11.6)	
32	55 (25.6)	5 (19.2%)	50 (26.5)	
64	27 (12.6)	0 (0.0)	27(14.3)	
128	8 (3.7)	0 (0.0)	8 (4.2)	
Tendency				0.007
Slice thickness (mm)	*n* = 251 (%)			
0.625	3 (1.2)	1 (3.4)	2 (0.9)	0.112
0.75	2 (0.8)	0 (0.0)	2 (0.9)	
1	19 (7.6)	1 (3.4)	18 (8.1)	
1.25	28 (11.2)	0 (0.0)	28 (12.6)	
1.5	11 (4.4)	0 (0.0)	11 (5.0)	
2	3 (1.2)	0 (0.0)	3 (1.4)	
2.5	5 (2.0)	1 (3.4)	4 (1.8)	
3	15 (6.0)	1 (3.4)	14 (6.3)	
4	1 (0.4)	0 (0.0)	1 (0.5)	
5	156 (62.2)	24 (82.8)	132 (59.5)	
6	2 (0.8)	0 (0.0)	2 (0.9)	
7	5 (2.0)	0 (0.0)	5 (2.3)	
8	1 (0.4)	1 (3.4)	0 (0.0)	
Tendency				0.012
Intravenous contrast	*n* = 252 (%)			
No	147 (58.3)	10 (34.5)	137 (61.4)	0.008
Yes	105 (41.7)	19 (65.5)	86 (38.6)	
